# Fostering accurate HIV/AIDS knowledge among unmarried youths in Cameroon: Do family environment and peers matter?

**DOI:** 10.1186/1471-2458-11-348

**Published:** 2011-05-19

**Authors:** Zacharie Tsala Dimbuene, Barthelemy Kuate Defo

**Affiliations:** 1 PRONUSTIC Research Laboratory and Department of Demography, University of Montreal, Quebec, Canada

## Abstract

**Background:**

The last three decades have seen a series of HIV interventions in sub-Saharan Africa. However, youths still have a mixture of correct and incorrect HIV/AIDS knowledge of transmission routes and prevention strategies. Previous studies have identified parents and peers as the most important socializing agents for youths. This paper assesses the relationships between family structure, family/peer communication about sexuality and accurate knowledge of transmission routes and prevention strategies.

**Methods:**

Data were drawn from the Cameroon Family Life and Health Survey (CFHS) conducted in 2002. The CFHS collected information on a representative sample of 4 950 people aged 10 years and over nested within 1 765 selected households from the 75 localities forming the administrative prefecture of Bandjoun, using detailed questionnaires about family, HIV/AIDS/STDs knowledge, sexual behaviors, contraception, health, media exposure, household assets and neighborhood characteristics. The survey cooperation rates were high (97%). For the purpose of this study, a sub-sample of 2 028 unmarried youths aged 12 - 29 years was utilized.

**Results:**

Overall, 42% of respondents reported accurate knowledge of documented HIV transmission routes whereas 21% of them had inaccurate knowledge such as AIDS can be transmitted through mosquito bites or casual contact with an infected person. Only 9% of respondents were knowledgeable about all HIV prevention strategies. Multivariate analyses showed that family structure, communication with parents/guardians and peers about sexual topics were significantly associated with accurate HIV knowledge. Additionally, age, education, sexual experience and migration had significant effects on accurate knowledge. Finally, living in poor households and disadvantaged neighborhoods significantly increased inaccurate knowledge of HIV transmission modes and prevention strategies.

**Conclusions:**

This paper evidenced the limited effects of HIV interventions/programmes in sub-Saharan Africa. Indeed, few respondents reported accurate knowledge about HIV transmission routes and prevention strategies. Findings showed that the role of family environment as source of accurate HIV knowledge transmission routes and prevention strategies is of paramount significance; however, families have been poorly integrated in the design and implementation of the first generation of HIV interventions. There is an urgent need that policymakers work together with families to improve the efficiency of these interventions. Peer influences is likely controversial because of the double positive effect of peer-to-peer communication on both accurate and inaccurate knowledge of HIV transmission routes.

## Background

Worldwide, population-based studies show that more than 90% of people ever heard of AIDS [1,2]. However, youths in Cameroon like other settings in sub-Saharan Africa (SSA) still have a mixture of beliefs and misconceptions about HIV transmission and prevention [3]. Indeed, few people report accurate HIV/AIDS knowledge. For example, the Cameroon Demographic and Health Survey (CDHS-2004) showed that, among the 15 - 29 years-old, 27% of females and 35% of males reported accurate HIV knowledge. Yet a better understanding of how HIV is transmitted or prevented is crucial for the disease control. Misconceptions can prevent individuals from making right choices, decisions and taking prevention strategies to protect themselves against AIDS.

Previous studies identified many sources of information about HIV/AIDS, including families, peers, schools, workplaces, communities, cultural belief systems and media [4-6]. Parents and peers emerged as the most important socializing agents [4,7,8]. The family environment provides structure and guidance to youths. Parents and other family members socialize youths, providing them with a system of values and norms. Additionally, peer-to-peer education was primarily used as a leading strategy for HIV interventions in SSA that aimed at increasing HIV/AIDS knowledge among adolescents and young adults [9].

Family environment, including family structure and parent/guardian-child communication about sexuality (hereafter PCC), is a key factor to cultivate and enhance HIV knowledge acquisition among youths. In this paper, PCC is expected to play an important role in fostering accurate knowledge of HIV transmission routes and prevention strategies. The roles of family figures in relation with HIV knowledge are discussed below.

### Parents

Generally speaking, two parents provide better guidance and teaching than do one-parent families [10]. They have the social power to teach children socially acceptable behaviors. With respect to sexual education, available evidence in SSA suggests that parents are often confronted with traditional values and norms regarding sexuality [11,12]. Most studies pinpointed the culture of silence as a strong barrier impeding parent-child discussions about sexuality [13,14]. Furthermore, parental involvement in sexual education is often gendered [15,16], with mothers being more involved than fathers [17]. Yet there is evidence that parents may be an important source of information about HIV/AIDS. A study in three urban sites, one in Dar-es-Salaam (Tanzania) and two in South-Africa (Cape Town and Mankweng) showed that parents are the preferred source of information about sexual topics [18]. However, parents are still understudied and underutilized in HIV interventions.

Parent-child communication (PCC) is the core of child socialization [15]: What are parents saying and are their children listening? Direct and open communication, and clear parent-child interactions are key components of knowledge acquisition processes which are associated with HIV knowledge [16]. Through sexual communication, parents instruct and transfer family values and norms about sexuality and behaviors to their children. A study in Nigeria showed that 74% of males and 60% of females had never talked with parents about sexual issues [19]. Notwithstanding these shortcomings, this study assumes that parent-child communication is positively (negatively) associated with accurate (inaccurate) HIV knowledge of transmission routes and prevention strategies.

### Older siblings, aunts, uncles and grandparents

Besides biological parents, other adults can inculcate normative patterns about sexual issues [17,20]. Their influences are likely stronger for youths who reside with them. For instance, grandmothers teach girls about sexuality and marriage [21]. Likewise, older siblings' influences were reported in Côte d'Ivoire [22]. In spite of the relative positive influences other adults may exert over adolescents and young adults, the presence of relatives within home can negatively impact accurate HIV knowledge if they send contradictory messages.

### Peer-to-peer communication

Previous research has shown that peer influences increase during adolescence and emerging adulthood [5,9,16,23-25]. Indeed, conversations with peers seem to be horizontal and more friendly [24], and facilitate easy talk about sexual-related topics. Because peer-to-peer communication about sexuality (hereafter PPC) is assumably associated with an increase in HIV knowledge [13,26], peer-educators were used as a leading strategy in HIV interventions in SSA. Although peer-educators are often well-trained, cultural norms still remain strong barriers for an easy talk about Reproductive Health (RH) matters. In practice, peer-educators may accordingly adapt their messages to avoid rejection by other peers and adults who disapprove of sexual education.

Associations between RH knowledge and sexual behaviors have been widely documented, assuming that an increase in knowledge leads to safer sexual behaviors [27-29]. There are also valuable reasons for which sexual behaviors can influence HIV knowledge [30-32]. Sexually experienced youths may be more prompt to seek information about AIDS because of potential vulnerability to AIDS due to unprotected sexual behaviors, whereas virgins may be distancing themselves from HIV/AIDS. Previous research showed that sexually experienced youths are more knowledgeable about HIV/AIDS compared with their sexually inexperienced counterparts [31,32]

Socio-demographic characteristics (age, education, gender, religious affiliation, migratory status) were found to correlate with HIV knowledge. Previous studies showed that HIV knowledge increases with age [32] and education [33]. Gender is another factor associated with HIV knowledge; males being more knowledgeable than females [34]. With respect to religion, youths attending liberal congregations like Protestants may be more knowledgeable compared with those from more conservative religious groups which are less open to RH matters [35]. Studies have also stressed migrants' vulnerability due to the family disruption that follows the decision to migrate [36]. However, there are two reasons for which migrants likely are more knowledgeable than non-migrants. First, migrants are often more educated than natives. Second, the rural-urban flows observed in SSA provide migrants with a greater media exposure in urban areas which can thereafter increases their knowledge about HIV/AIDS.

Higher socioeconomic statuses at household and community levels increase the accuracy of HIV knowledge [37,38]. Indeed, youths from better-off households are likely to reside with better-educated parents who feel more comfortable to communicate about HIV/AIDS. Parental education provides additional skills and competences to teach children about HIV/AIDS [39,40]. On the other hand, youths from disadvantaged neighborhoods are often less educated and have limited access to media. Hence, they are likely to report inaccurate knowledge about HIV/AIDS. Finally, previous research stressed urban-rural differences about HIV knowledge; urban residents being more knowledgeable about HIV/AIDS than their rural counterparts [3].

Based on previous research, the present study is aimed at assessing the effects of family environment and peers in fostering accurate knowledge of HIV/AIDS transmission and prevention among unmarried youths for whom family/peer influences on RH matters remain socially relevant.

## Methods

### Setting

Cameroon is often referred to as Africa in *miniature *due to geographic, climatic and ethnic diversities (with more than 200 ethnic groups). In contrast, Bandjoun which is situated in the grass-fields of the West region of Cameroon is culturally homogeneous. The majority of the population is of the *Bamiléké ethnic group*, the largest ethnic group in Cameroon. Bandjoun is an area of 274 square kilometers and is representative of the belief system, customs and social structure of the population of West Cameroon [41]. It combines the features of a highly modernized environment with a typical traditional Cameroonian society. The urban and semi-urban localities have one of the country's universities, three public hospitals, two private hospitals in operation since the early 1950s, about a dozen public health centers, several traditional healers attracting people from various social strata, several high schools, and infrastructures for communication and transportation. The large number of schools in this setting is partly a result of the secular importance the local population attaches on their children's education. Compared with the rest of the country, the western part of Cameroon including Bandjoun is more educated. In the rural areas of Bandjoun, there are over 70 chiefdoms with traditional authorities and practices, an extensive practice of polygamy, agricultural production, and extensive farming. The geographical distribution of the population reflects one of the highest population densities, with a population of 51 624 inhabitants and a density of 188 inhabitants per km^2 ^[42].

With respect to sexual education, sexuality is a taboo topic in Bandjoun like in many traditional societies in Cameroon [11]. In fact, parents tend to remain silent when it comes the time to teach children about RH issues, a result of the socio-cultural environment and certain traditional practices of Africa and Cameroon in particular. Such context likely hampers the role parents can play to teach children about sexual matters.

### Study participants and sampling design

Data are drawn from a random and representative sample of the Cameroon Family and Health Survey carried out in 2002 (CFHS-2002), under the auspices of the Population Observatory in Socio-clinical Epidemiology (POSE) in Bandjoun. An updated distribution of the population based on the 1987 Cameroonian census was used to ensure the representativeness of the sample; and the 75 localities forming this area were included in the survey [41]. Within each locality, all households were listed. Each locality used probability samples in which all households have a nonzero chance of inclusion. Proportionate numbers of households were selected at random from the list of all given households within each locality, resulting of 1 765 selected households. When selected, all individuals aged 10 years and above within the household were interviewed. A total of 4 950 individuals of both sexes were interviewed in the detailed questionnaire using forced-choice questions and face-to-face technique. Cooperation rates were very high. Indeed, of 5 293 eligible people in the selected households, 4 950 individuals participated in the survey leading to a total response rate of 97%. For the purpose of this study, 2 028 unmarried adolescents and young adults aged 12 - 29 years was used (thereafter, referred to as youths) [43]

The survey measures were specifically designed for this study after an extensive review of the literature and taking into account the social, cultural, and societal differences. Validated instruments from national and international surveys about reproductive health matters were used. The questionnaire was pre-tested prior to data collection, and translated in the local language. In addition to a demographic section, the questionnaire consisted of five sections: sexuality and fertility, contraceptive knowledge and use, knowledge about HIV/AIDS and other STDs, family environment, and media exposure. Sociodemographic characteristics sought included age, gender, marital and migration status, urban/rural location, and education. Participants were asked questions regarding knowledge and beliefs about HIV/AIDS including modes of HIV transmission, prevention strategies against HIV, and sexual behavior.

Trained and well-educated interviewers administrated detailed individual questionnaires in local language about family, sexual behaviors, contraception, HIV/AIDS/STDs, health, and media exposure. Besides the detailed individual information about RH matters, two other questionnaires based on international instruments such as DHS were used to collect household and neighborhood characteristics. Sample questions about household characteristics included possessions (e.g., electricity, radio, TV, refrigerator, cleaned water) and materials (e.g., walls, roof, floor). Neighborhood-level relevant information consisted of main access road in the locality, economic activities and other commodities such as telephone, market, and school.

## Measures

### Dependent variables: Accurate vs. inaccurate knowledge of HIV transmission routes, and knowledge of HIV prevention strategies

Most studies have utilized scales to measure HIV knowledge [27,32]. However, after three decades of HIV interventions, there are empirical and programmatic reasons to examine whether people have a thorough knowledge of HIV transmission routes and prevention strategies. To answer this question, a restrictive operationalization was adopted: each respondent must report correct answers about all items. HIV knowledge included *yes/no *items on how AIDS is transmitted (6 items) or prevented (8 items). The transmission routes consisted of 4 items pertaining to accurate HIV knowledge (or true/probable transmission routes) and 2 items pertaining to inaccurate HIV knowledge (or improbable transmission routes). Accurate knowledge of HIV transmission routes is a binary response variable coded 1 if youths reported four correct responses and 0 otherwise. Likewise, inaccurate knowledge is coded 0 if youths rejected the two misconceptions and 1 otherwise.

HIV prevention strategies include items about "How AIDS can be prevented?" Eight items were listed but interviewers could not prompt respondents. Accurate knowledge of HIV prevention strategies is coded 1 if respondents reported eight correct responses and 0 otherwise. Cronbach's alpha was used to assess the reliability (internal consistency) for the two constructs. The two scales showed adequate reliability (α Cronbach = .74 and .81 for HIV transmission and prevention, respectively).

### Key independent variables

#### Family structure (FS)

This variable is assessed using a set of *yes/no *questions: "With whom do you live at the time of the survey?" Responses include father, mother, brother/sister, cousins, uncle/aunt, grandfather/grandmother, friend, playmate, institution, or alone. The last four items (friend, playmate, institution, or alone) consisted of few cases and were thereafter excluded. Six mutually exclusive categories of family structures were built: *two biological parents*; *father-only*; *mother-only*; *brother/sister/cousin*; *uncles/aunts*; and *grandparents*.

#### Family/peer communication

Knowledge acquisition is a dynamic process. For Piaget [44], knowledge is neither innate nor contained in objects. In contrast, it is dynamically created by interactions between the human and these objects. Thus, knowledge is considered as an active and operative assimilation. Hence, the relevant period of life that might be considered to address the influences of family/peer communication on HIV knowledge is important. To account for the cumulative property of knowledge acquisition, the paper builds an index including communication at age 12 and at the time of the survey. Parent-child (*PCC*) and peer-peer communication (*PPC*) consisted of five topics in *yes/no *formats: puberty, sexual education, IST/HIV/AIDS prevention, pregnancy prevention, and alcohol/drugs. Responses were summed in two scales of PCC and PPC ranging from 0 to 10 (α Cronbach = .94 and .95, respectively).

### Other correlates at the individual, familial and community levels

Individual characteristics included gender, age, education (in completed years), ever had sexual intercourse, migration, and religion. Family-level variables include the household socioeconomic status (HHSES) and parent/guardian's education. Neighborhood-level variables include the community development index (CDI) and place of residence.

HHSES was constructed using household's possessions (electricity, radio, TV, refrigerator, bicycle, land ownership, livestock and commercial goods), main source of drinking water, type of toilet facilities, and household's materials (walls, roof and floor). Likewise, CDI consisted of main access roads, economic activities (agriculture, livestock, fishing, commerce, manufacturing/handicraft) and other commodities (telephone, schools, local market, post office, recreational centre, public transportation, movies centre, hospital, and youth centre). Principal component analysis (PCA) was used to build HHSES (13 items) and CDI (18 items). PCA transforms linearly an original set of variables into a substantially smaller, more coherent set of uncorrelated variables (or *factors*) to solve multicollinearity threats among initial variables.

## Statistical Methods

Firstly, the relationships between family structure, PCC, PPC and knowledge of HIV transmission routes are examined. Because previous research reported a correlation between accurate and inaccurate knowledge of HIV transmission routes [32,45], using two independent equations is inefficient because it ignores this correlation; therefore, bivariate probit models are preferred [46]. The formulation of bivariate probit models is as follows.

The ACCURATE equation is:(1)

where FS, PCC and PPC are the key independent variables, Z a vector of other factors, and the disturbance term. The probability of accurate knowledge of HIV transmission routes can be computed as follows:

Similarly, the INACCURATE equation is:(2)

where *ε*_*I *_is the disturbance term. The disturbances terms (*ε*_*A *_and *ε*_*I*_) are assumed to have a bivariate normal distribution (BVN) with a mean vector and covariance matrix. The parameter ρ captures the correlation between accurate and inaccurate knowledge of HIV transmission routes. Under the null hypothesis (*ρ *= 0), two independent probit equations provide unbiased estimates. The bivariate probit coefficients *β*_*A *_and *β*_*I *_provide a suitable means of comparing the influences of FS, PCC, PPC, and other correlates under the alternative hypothesis *ρ *≠ 0.

Secondly, logistic regression was used to estimate the effects of FS, PCC and PPC on accurate knowledge of HIV prevention strategies. The reference category for a variable has an estimated coefficient of zero (or alternatively an OR = 1). An estimated coefficient greater than zero (less than zero) indicates a positive (or negative) association with accurate knowledge of HIV prevention strategies and is associated with an OR greater (or lesser) than unity.

Another methodological issue that needs to be addressed relies on the hierarchical nature of data. Because information on youths was collected from sampled households, youths are not independent observations. This also suggests that youths can share common household characteristics. It is, therefore, necessary to account for the correlation of observations so as to derive "true" estimates of standard errors and other associated values including p-value and confidence intervals. It is possible to control for the correlation of observations for the same household by using the appropriate options in the STATA software [47,48]. In STATA, when the cluster option is specified, it then corrects for the fact that youths of the same household are not independent observations. This yields robust standard errors, those obtained via the Huber-White sandwich estimator of variance. Moreover, should there exist intra-household correlation, the robust standard errors are better indicators of the sample-to-sample variability of the parameter estimates and therefore produce more accurate tests of the effects of covariates [47].

### Ethical considerations

Ethical approval for the project and surveys from which data used in the paper are drawn was obtained from the National Ethics Committee of Cameroon (Yaoundé, Cameroon) and the Ethics Committee of the University of Montreal (Montreal, Canada). Additionally, strict confidentiality was maintained about the identity of respondents due to the sensitivity of RH matters among unmarried youths. In fact, interviewers were instructed to respect respondents' privacy during and after the interview. Specifically, interviews were not conducted in the presence of parents or guardians.

## Results

### Descriptive results

Table 1 describes the selected independent variables into three blocks: individual factors, family environment and community characteristics. The sample consisted of 54% female youths and a mean age of 16.5 years (Range: 12 - 29 years). Most of them completed primary school (*M *= 8.1; *SD *= 2.9), nearly half are non-native (49%), are mostly Catholics whereas one-third of youths ever had sexual intercourse. As regards to family structure, 48.7% of youths lived with two biological parents; a total of 22% was living with one biological parent, mostly the mother (17%). Nearly 10% was living with brother/sister/cousin, and 6% lived with other relatives (aunts/uncles). Another important feature is the higher proportion of youths living with grandparents (14%). One-third of respondents live in poor households. Parents' education is relatively high; only 30% of parents were illiterate, whereas others reported primary (40%) or secondary education or higher (30%). As expected, the mean score of PCC is low (*M *= 1.566; *SD *= 2.019), while the levels of PPC are moderate (*M *= 2.142; *SD *= 2.259). Finally, 17% of youths live in urban areas, and one-quarter of them reside in poor neighborhoods.

**Table 1:** Descriptive statistics of participants

Variables	% or Mean ± SD (Sample size: *N *= 2 028)
**Individual factors**	
Gender Male	45.9
Age (in years) [range 12 - 29]	16.5 **± **4.2
Education (in years completed) [Range 0 - 24]	8.1 **± **2.9
Sexually experienced (% yes)	32.0
Migratory status (% migrant)	48.5
Religion	
Catholics	68.1
Protestants	26.3
Other	5.6
**Family environment**	
Family structure	
Two-parent	48.7
Father-only	5.0
Mother-only	17.2
Brother/Sister	9.5
Uncle/Aunts	5.6
Grandparents	14.0
HWI (40% poorest)	29.9
Parent/guardian-child communication [Range 0 - 10]	1.566 **± **2.019
Education of parent/guardian	
None	30.3
Primary	40.2
Secondary & +	29.5
**Peer influences**	
Peer-to-peer communication [Range 0 - 10]	2.142 ± 2.259
**Community characteristics**	
CDI (40% poorest)	25.0
Urban Residence	17.4

Source: CFHS (2002)

Table 2 describes the phrasing of knowledge of HIV transmission routes and prevention strategies. A significant proportion of youths know how HIV is transmitted (Panel A.1 of Table 2): unprotected sexual intercourse (88.3%), sharing needles or unclean medical equipment (69.2%), blood transfusions (74.1%) and mother-to-child transmission (54.6%). Taken together, 41.6% of youths report accurate knowledge of HIV transmission routes. Likewise, 8% of youths have heard of HIV but couldn't enumerate any transmission route. One-fifth of youths think that AIDS is transmitted through mosquito bites and/or causal contacts (Panel A.2 of Table 2).

**Table 2:** HIV/AIDS Knowledge among adolescents and young people in Bandjoun (Cameroon)

Variables	Range	Number of items	Cronbach's alpha	Correct answer	Frequency of correct (or incorrect) answers	%
**A. Knowledge of HIV transmission routes**	0-6	6	.74			
**A.1 Accurate knowledge of HIV transmission routes**	0-4	4				
Unprotected sexual intercourse				Yes	1 791	88.3
Sharing needles/unclean medical equipment				Yes	1 403	69.2
Blood transfusions				Yes	1 503	74.1
Mother-to-child transmission				Yes	1 107	54.6
% Accurate knowledge of HIV transmission routes						41.6
**A.2 Inaccurate knowledge of HIV transmission routes**	0-2	2				
Mosquito or other insect bites				No	296	14.6
Casual contact with infected person				No	201	9.9
% Inaccurate knowledge of HIV transmission routes						21.1
**B. Knowledge of HIV prevention strategies**	0-8	8	.81			
Sexual abstinence/chastity				Yes	1 543	76.1
Stay faithful to partner				Yes	1 186	58.5
Encourage partner to stay faithful				Yes	898	44.3
Avoid contaminated blood transfusions				Yes	984	48.5
Use condoms correctly at every sexual intercourse				Yes	1 245	61.4
Avoid not sharing needles				Yes	1 006	49.6
Avoid commercial sex workers				Yes	748	36.9
Avoid casual partners				Yes	320	15.8
% Accurate knowledge of HIV prevention strategies						9.0

Source: CFHS (2002)

Knowledge of HIV prevention strategies showed greater variations (Panel B of Table 2). Correct answers were as follows: sexual abstinence/chastity (76.1%), condoms (61.4%), faithful to partner (58.5%), avoid sharing needles (49.6%), avoid blood transfusions (48.5%), encourage partner to stay faithful (44.3%), avoid commercial sex workers (36.9%) and casual sexual partners (15.8%). Close to 8% of young people do not know any HIV prevention strategies. Overall, less than one tenth (9%) reported satisfactorily eight HIV prevention strategies.

### Multivariate results

#### Co-occurrence of accurate and inaccurate knowledge of HIV transmission routes

Table 3 presents estimated coefficients from bivariate probit models. The gross effects allow determining the direct effect of FS, PCC and PPC. Models 1 and 2 report the effects of FS controlling for PCC and PPC, respectively. Model 3 displays bivariate probit coefficients for all selected variables.

**Table 3:** Estimated coefficients of bivariate probit models for accurate and inaccurate knowledge of HIV transmission routes among young people in Bandjoun (Cameroon)

	Gross effects		Model 1		Model 2		Model 3	
	
Variables	Accurate	Inaccurate	Accurate	Inaccurate	Accurate	Inaccurate	Accurate	Inaccurate
**Family environment**								
Family structure (Ref.: *Grandparents*)								
Two-parent	.201**(.085)	-.147(.089)	.175**(.087)	-.169*(.092)	.193**(.087)	-.160*(.092)	.138**(.069)	-.146(.102)
Father-only	.387***(.107)	-.005(.114)	.388**(.148)	-.006(.156)	.328**(.146)	-.024(.156)	.226*(.121)	-.041(.168)
Mother-only	.175*(.099)	-.314**(.110)	.119(.103)	-.369**(.114)	.138(.102)	-.359**(.114)	.065(.109)	-.297**(.119)
Brother/Sister	.329**(.114)	-.212*(.126)	.338**(.119)	-.193(.129)	.367**(.119)	-.179(.129)	.204**(.099)	.171(.136)
Uncle/Aunts	.174(.139)	-.326**(.159)	.132(.145)	-.409**(.165)	.151(.144)	-.399**(.165)	.115(.151)	-.378**(.170)
HWI (Ref.: *middle/rich*)	-.238***(.048)	.078(.053)					-.162**(.069)	.069(.072)
Parent/guardian-child communication	.118 ***(.014)	-.041**(.014)	.116***(.015)	-.041**(.016)			.048**(.018)	-.039**(.019)
Education of P/G (Ref. : *none)*								
Primary	.104(.065)	.016(.071)					.086(.074)	.047(.081)
Secondary & +	.192**(.068)	-.149*(.077)					.112*(.061)	-.102(.093)
**Peer influences**								
Peer-to-peer communication	.081***(.012)	.022(.014)			.079***(.013)	.024*(.014)	.066**(.032)	.007(.017)
**Individual factors**								
Gender Male (Ref.: *Female*)	-.056(.056)	.027(.063)					.023(.045)	.021(.064)
Age (in years)	.112***(.002)	.040*(.021)					.052***(.011)	.021*(.011)
Education (in years completed)	.105***(.008)	-.095**(.048)					.082**(.013)	-.053***(.013)
Sexually experienced (Ref.: *NO*)	.413***(.045)	.141**(.049)					.103**(.041)	.134*(.068)
Migratory status (Ref.: *non-migrant*)	.166***(.044)	.084*(.049)					.043*(.024)	.039(.068)
Religion (Ref.: *Catholic*)								
Protestants	.030 (.049)	-.029(.055)					.021(.068)	-.025(.074)
Other	-.046(.089)	-.025(.099)					-.025(.130)	-.036(.139)
**Community characteristics**								
CDI (Ref. : *middle/rich*)	-.084*(.051)	.143**(.058)					-.051(.071)	.111**(.052)
Type Residence (Ref. : *rural*)	.127**(.058)	-.162**(.062)					.086*(.048)	-.152**(.067)
ρ (Rho)	n/a		.238***		.235***		.261***	
Log- likelihood	n/a		-2 351.814		-2 366.474		-2 224.502	

Source: CFHS (2002) Models 1: Family structure + parent-child communication; Model 2: Family structure + peer-to-peer communication; Model 3: Full model. *** *p *< 0.01 (two-tailed test); ** *p *< 0.05 (two-tailed test); * *p *< 0.10 (two-tailed test)

The parameter of primary interest is the correlation between the disturbance terms (*ε*_*A *_and *ε*_*I*_) in equations (1) and (2). It measures the extent to which accurate and inaccurate knowledge of HIV transmission routes are correlated after controlling for observed factors such as FS, PCC, PPC, and other characteristics. Results show a positive and significant correlation. It means that youths who, for reasons not observed in the data, report accurate knowledge of HIV transmission routes are also more likely to report higher levels of misconceptions. In other words, factors shaping true or false beliefs about AIDS in this setting are correlated. For instance, if people within the neighborhood believe that AIDS is a God's curse or punishment, youths will be likely to misbelieve in this way, rather than seeking for accurate information about the disease.

Associations between the key independent variables (FS, PCC and PPC) and knowledge of HIV transmission routes showed interesting results. The gross effects of FS (Table 3; Column 2) indicate that, living with one or two parents, with brother/sister significantly increases the likelihood of reporting accurate knowledge of HIV transmission routes, compared to those living with grandparents. In contrast, living with mother, brother/sister or with uncles/aunts significantly decreased the likelihood of inaccurate knowledge of HIV transmission routes.

As expected, PCC significantly increases (or decreases) accurate (inaccurate) knowledge of HIV transmission routes. Likewise, PPC is positively and significantly associated with accurate knowledge of HIV transmission routes; whereas it showed an insignificant positive effect with inaccurate knowledge. This finding means that peers may likely convey contradictory messages about HIV transmission.

At individual level, age, education, sexual experience, and migration are significantly associated with accurate knowledge of HIV transmission routes (*p *< 0.01). Interestingly, education decreased significantly the likelihood of inaccurate knowledge of HIV transmission routes. Three other variables (age, being sexually experienced and migration) showed positive and significant effects on inaccurate knowledge of HIV transmission routes.

Findings indicate that living in poor households and disadvantaged neighborhoods has negative and significant effects on accurate knowledge of HIV transmission routes. Living with better-educated parents and urban residence significantly increase accurate knowledge of HIV transmission routes. These factors show a marginal negative effect on inaccurate knowledge of HIV transmission routes (*p *< 0.10).

From Model 1, the effects of FS decreased slightly when PCC and PPC are included but did not substantially modify the statistical significance. Model 3 confirms the effect of FS, PCC, and PPC on accurate knowledge of HIV transmission routes. In addition, the effects of individual factors such as age, education, and sexual experience remained statistically significant. Models 1 - 2 about inaccurate knowledge of HIV transmission routes showed similar results as previously observed. Finally, Model 3 showed that living with mother or with uncles/aunts, ever talked with parents/guardians about sexuality, education, and living in urban areas decrease significantly the likelihood of inaccurate knowledge of HIV transmission routes. In contrast, living in disadvantaged neighborhoods has a positive and significant effect on inaccurate knowledge of HIV transmission routes.

#### Knowledge of HIV prevention strategies

Table 4 displays logistic coefficients of the effects of FS, PCC, PPC, and other correlates on the accurate knowledge of HIV prevention strategies. Models 1 and 2 assess the effects of FS accounting for PCC and PPC. Finally, Model 3 controls for other individual, household and community factors. Findings show that FS, PCC and PPC significantly increased the likelihood of accurate knowledge of HIV prevention strategies. Living in two-parent (*β *= .160; *p *< 0.05) or father-only families (*β *= .664; *p *< 0.05), or living with brother/sister (*β *= .469; *p *< 0.05) are associated with higher likelihoods of accurate knowledge of HIV prevention strategies. Likewise, PCC (*β *= .286; *p *< 0.01) and PPC (*β *= .234; *p *< 0.01) significantly increase the knowledge of HIV prevention strategies.

**Table 4:** Logistic coefficients of HIV/AIDS knowledge of prevention strategies among young people in Bandjoun (Cameroon)

Variables	Gross effects	Model 1	Model 2	Model 3
**Family environment**				
Family structure (Ref.: *Grandparents*)				
Two-parent	.160**(.071)	.157**(.69)	.147**(.65)	.073**(.35)
Father-only	.664**(.234)	.480**(.245)	.559**(.221)	.359**(.159)
Mother-only	.225(.166)	.106(.169)	.142 (.164)	.102 (.131)
Brother/Sister	.469**(.191)	.362**(.189)	.421**(.198)	.227**(.101)
Uncle/Aunts	.264(.229)	.185(.242)	.151 (.237)	.191 (.213)
HWI (Ref.: *middle/rich*)	-.525***(.103)			-.217**(.112)
Parent/guardian-child communication	.286***(.025)	.283***(.025)		.115***(.029)
Education of P/G (Ref. : *none)*				
Primary	.208**(.099)			.240*(.129)
Secondary & +	.293**(.104)			.189*(.084)
**Peer influences**				
Peer-to-peer communication	.234***(.022)		.232***(.023)	.108***(.027)
**Individual factors**				
Gender Male (Ref.: *Female*)	-.217**(.091)			-121(.102)
Age (in years)	.143***(.012)			.089**(.039)
Education (in years completed)	.254***(.019)			.160***(.024)
Sexually experienced (Ref.: *NO*)	.930***(.098)			.275**(.134)
Migratory status (Ref.: *non-migrant*)	.217**(.110)			.137(.108)
Religion (Ref.: *Catholic*)				
Protestants	.091(.104)			.122(.117)
Other	.063(.201)			.014(.225)
**Community characteristics**				
CDI (Ref. : *middle/rich*)	-.315**(.107)			-.131**(.058)
Type Residence (Ref. : *rural*)	.387**(.118)			.320**(.131)
Log- likelihood	...	-1 285.056	-1 294.152	-1 181.146

Source: CFHS (2002) Models 1: Family structure + parent-child communication; Model 2: Family structure + peer-to-peer communication; Model 3: Full model. *** *p *< 0.01 (two-tailed test); ** *p *< 0.05 (two-tailed test); * *p *< 0.10 (two-tailed test)

Also, age, education, sexual experience, migratory status, parent/guardian's education, and place of residence significantly increased the odds of accurate knowledge of HIV prevention strategies. Surprisingly, gender male showed negative and significant effect (*β *= -.217; *p *< 0.05). Living in poor households (*β *= -.525; *p *< 0.01) and disadvantaged neighborhoods (*β *= -.315; *p *< 0.05) significantly decrease the odds of accurate knowledge of HIV prevention strategies.

From Models 1 - 2 (Table 4), FS shows significant direct and indirect effects on accurate HIV knowledge of prevention strategies. Furthermore, PCC and PPC significantly increase the odds of accurate knowledge of HIV prevention strategies. In Model 3, findings show that a number of factors increased or decreased the odds of accurate knowledge of HIV prevention strategies. For instance, living with two biological parents, with a biological father or with brother/sister/cousins in one hand, and on other hand, PCC and PPC are associated significantly with higher likelihood of accurate knowledge of HIV prevention strategies.

Age, education, sexual experience and migratory status appeared as strong predictors of accurate knowledge of HIV prevention strategies. Youths in urban areas are significantly more knowledgeable about HIV/AIDS prevention strategies than their rural counterparts. As expected, poor households and disadvantaged neighborhoods show negative and significant effects on accurate knowledge of HIV prevention strategies.

## Summary and discussion

The study examined the associations between family structure, family/peer communication about sexuality, and other correlates and accurate (inaccurate) transmission routes and prevention strategies about HIV/AIDS. The proportion of respondents who reported a thorough knowledge of HIV transmission routes and preventive strategies was very low in this sample like at the national level [49]. For instance, less than half of respondents are knowledgeable about the four documented HIV transmission modes. One fifth of respondents misbelieve that AIDS can be transmitted through mosquito bites and/or casual contact with an infected person. This proportion is quite lower when considering HIV prevention strategies. Less than one-tenth reported accurate knowledge of the eight HIV prevention strategies. Our findings suggest the persistence of a wide window of prevention efforts three decades after the first generation of HIV interventions were implemented in SSA.

These findings have policy implications and fit with the research question addressed in this paper because the first generation of HIV interventions mainly focused on individuals; therefore neglecting the daily-life environment. Although peer influences have been widely documented in HIV interventions in SSA, parents and more largely families has been addressed only recently [50]. Yet parents and other family members play an important role in sexual education even though they can feel uncomfortable to talk with their children about sexual topics [14]. The main findings are that youths significantly and concurrently report accurate and inaccurate knowledge of HIV transmission modes [32], and family structure as well as family and peer-to-peer communication are key explanatory variables on which HIV educational campaign efforts should be directed at, among other putative factors identified here and documented in previous studies (age, education, sexual experience, migration, HHSES, and CDI).

Net of controls, family structure showed significant effect on knowledge of HIV transmission routes and prevention strategies. This paper provides new insights that parents may act as sexual educators as reported in recent studies [51]. Although descriptive findings resulted in low levels of PCC, this variable showed a positive and significant effect on accurate knowledge of HIV transmission routes and HIV prevention strategies. In other words, "*little but does much*". From a programmatic feature, the challenge is not only to increase communication about sexuality between parents and children but first and foremost, to establish or provoke this communication to break the cultural silence and discomfort that may hinder parental influences.

Of interest in the paper was also the association between PPC and accurate HIV knowledge. It was found that PPC increased significantly the probability of accurate knowledge of HIV transmission modes and prevention strategies [24]. This is in line with previous research [26], and at least explains why peer-to-peer strategy was widely used in HIV interventions in SSA. However, further research is needed to understand the content of messages peers convey due to its *double positive *effect on accurate and inaccurate knowledge of HIV transmission routes. In other words, the accuracy and appropriateness of peers' knowledge about HIV transmission routes or prevention strategies are questionable. Therefore, more guidance is needed for youths to use HIV information from peers appropriately. Here again, parents and other family parents, including older siblings, aunts/uncles, and grandparents as well may be influential and helpful to correct the information if necessary.

Previous HIV interventions posited that increasing HIV knowledge leads to prevention strategies, such as condom use. This paper was interested on the association between sexual behavior and accurate HIV knowledge. Results indicated a strong and significant association. These findings mean that youths react differently to HIV prevention campaigns depending upon their sexual experience. One possible explanation is that virgin youths are distancing themselves from HIV/AIDS because they feel invulnerable to AIDS due to their sexual inexperience [30]. These attitudes may jeopardize them when it comes the time to start sexual life if they are less knowledgeable about HIV transmission routes and prevention strategies.

The role of socioeconomic disadvantage was also examined. Living in poor homes and disadvantaged neighborhoods significantly decreased the likelihood of accurate knowledge about HIV/AIDS. At the same time, economic hardship within home or neighborhood increased misconceptions about HIV transmission routes. The broad array of factors related to socioeconomic deprivation, like a low media exposure or lower levels of educational attainment may explain the negative effect of poor homes and disadvantaged neighborhoods on accurate knowledge of HIV/AIDS among youths in this setting [3].

### Methodological considerations

While the efforts were taken to ensure the representativeness of the sample in the study, the fact that respondents were recruited from a homogeneous ethnic setting (98% of inhabitants belong to the same ethnic group) limits our ability to generalize the findings to youths in other regions of Cameroon. In addition, it is impossible to assess causation because data are cross-sectional. This can also explain the small variation observed in terms of parent-child communication about sexuality. Yet, information at national level should provide different patterns of family environment or educational attainment among youths. Because knowledge acquisition is a long-lasting process, longitudinal studies are recommended for future research to capture causality and dynamic aspect of HIV knowledge and the developmental stages of youths.

An important methodological issue considered in this paper is the correlation between accurate and inaccurate knowledge about HIV transmission routes. Previous studies have raised this question [32], but they have not accounted for this correlation in subsequent analyses. That may lead to biased estimates of the correlates of HIV knowledge. In considering bivariate probit models to estimate simultaneous equations, this paper further our understanding of the effects of family environment and peers on accurate (and inaccurate) knowledge about HIV transmission routes. This statistical technique requires no particular restriction about the sample size which is quite large in this study. The fact that accurate and inaccurate knowledge about HIV transmission in this sample are positively and significantly correlated has policy implications. Policymakers should emphasize distinctively documented and undocumented (or improbable) HIV transmission modes in RH interventions to reduce or eliminate misconceptions about HIV/AIDS among youths in this setting and at national level as well.

Notwithstanding the restrictive conceptualization of HIV knowledge and the empirical contribution of the paper, there are a number of potential limitations that need to be addressed. Firstly, the content, frequency and comfort of parent-child communication were not examined here because there were no questions in the survey about these topics [16,24]. Yet they may be more important than communication *per se*. With regard to the measurement of sexual communication for both parents/guardians or peers, the *yes/no *items used in the survey instruments are likely a source of the small variation observed in the data. Secondly, parents' knowledge of HIV/AIDS was not measured. In this case, the findings were based on an implicit hypothesis which assumes that parents are sufficiently "knowledgeable about HIV/AIDS" to teach their children. Future research needs to use a direct measure of parents' knowledge about HIV/AIDS to be compared with youths. In fact, a direct correlation between parental and youth HIV/AIDS knowledge is the more appropriate manner to test how parents/guardians can foster accurate knowledge about HIV transmission routes and prevention strategies among their children.

## Conclusion

Our findings suggest that family environment is an important component of HIV interventions in SSA. On this debate, the paper is confirmatory and exploratory. It evidenced the role of parents as sex educators, provided insights that efficient HIV youth-oriented interventions must be rooted in their contexts (families, peers, schools, neighborhoods) [52]. Thus, this research is confirmatory as such. Against the dominant discourse in SSA claiming that parents do not teach their children about sexuality, the study showed that both family structure and parent-child communication are significant predictors of accurate HIV knowledge. Programmatically, parents can likely do better if they are well-equipped and trained to teach children. Therefore, family environment must be a key factor in the design and implementation of HIV interventions targeted to youths.

From an exploratory point, this paper can serve as a first step; no study to date has addressed the interplay between family environment and HIV knowledge in SSA. Previous research has documented the barriers which hamper parents/guardians to act as sex educators in SSA. Discomfort and the culture of silence according which sexuality is a taboo topic are the most cited barriers. This is not a specific pattern of SSA countries as discomfort has been documented even in other developing countries [6]. Policymakers have to work together with families to find a way to overcome these barriers.

The role of peers deserves a particular attention. Peer-to-peer strategy has been widely utilized in HIV interventions in SSA to enhance HIV knowledge among young people. However, our findings reported a double positive effect of peer-to-peer communication on HIV transmission routes. This result appeals to additional scrutiny about the contents of messages peers convey. In fact, if the information they convey are fully true, one should expect a positive (negative) association between PPC and accurate (inaccurate) knowledge of HIV transmission modes. This was not the case in the paper.

## Competing interests

The authors declare that they have no competing interests.

## Authors' contributions

All authors (ZTD, BKD) participated in the conception of research questions and study design. BKD was the principal investigator of Cameroon Adolescent Reproductive Health Program (CAREH) which consisted of two components, including Intervention and Research. ZTD was responsible for analyzing data and drafting the paper. All authors read and approved the manuscript.

## Pre-publication history

The pre-publication history for this paper can be accessed here:

http://www.biomedcentral.com/1471-2458/11/348/prepub
